# Immune and stromal tumour biology associated with neoadjuvant radiotherapy response in locally advanced rectal cancer

**DOI:** 10.1038/s41416-026-03575-y

**Published:** 2026-08-18

**Authors:** Lily V. S. Hillson, Ashley K. McCulloch, Ross K. McMahon, Phimmada Hatthakarnkul, Aula Ammar, Timothy S. Maughan, Enric Domingo, Sudhir B. Malla, Raheleh Amirkhah, James H. Park, Joanne Edwards, Philip D. Dunne, Sean M. O’Cathail, Campbell S. Roxburgh

**Affiliations:** 1 Wolfson Wohl Cancer Research Centre, School of Cancer Sciences, University of Glasgow, Bearsden, UK; 2 Academic Unit of Surgery, School of Medicine, University of Glasgow, Glasgow, UK; 3 Department of Oncology, Medical Sciences Division, University of Oxford, Old Road Campus Research Building, Oxford, UK; 4 Patrick G. Johnston Centre for Cancer Research, Queens University Belfast, Belfast, Northern Ireland UK; 5 Department of General Surgery, Queen Elizabeth University Hospital, Glasgow, UK; 6 Cancer Research UK Scotland Institute, Glasgow, UK

**Keywords:** Colon cancer, Radiotherapy

## Abstract

**Background:**

Response to neoadjuvant chemoradiotherapy in locally advanced rectal cancer (LARC) is heterogeneous. The Neoadjuvant Rectal (NAR) score quantifies treatment response via tumour downstaging and nodal status and correlates with survival outcomes. This work set out to explore whether low NAR and high NAR tumours represent biologically distinct groups pre-treatment.

**Methods:**

Transcriptome microarrays quantified RNA expression within pre-treatment biopsies from LARC patients in GSE87211 and S:CORT consortium Grampian datasets. A validation cohort from the Beatson West of Scotland Cancer Centre was analysed using multiplex immunofluorescence and H&E-derived tumour-stromal ratios.

**Results:**

Seventeen MSigDB Hallmarks were enriched in high NAR tumours; ‘Epithelial-Mesenchymal Transition’ was notably enriched (padj < 0.001). Immune-related signatures were enriched in low NAR tumours (*p* < 0.25). Both cohorts showed higher Microenvironment Cell Population counter ‘Cytotoxic Lymphocytes’ in low NAR (*p* < 0.05) and more stromal cells (‘Endothelial Cells’, ‘Fibroblasts’; *p* < 0.05) in high NAR tumours. CD8 densities confirmed higher immune infiltration in low NAR tumours.

**Discussion:**

Pre-treatment biopsies show consistent microenvironmental differences between high and low NAR tumours. While immune and stromal features are associated with response, the observed differences are insufficient to serve as standalone predictive biomarkers.

**Figure:**
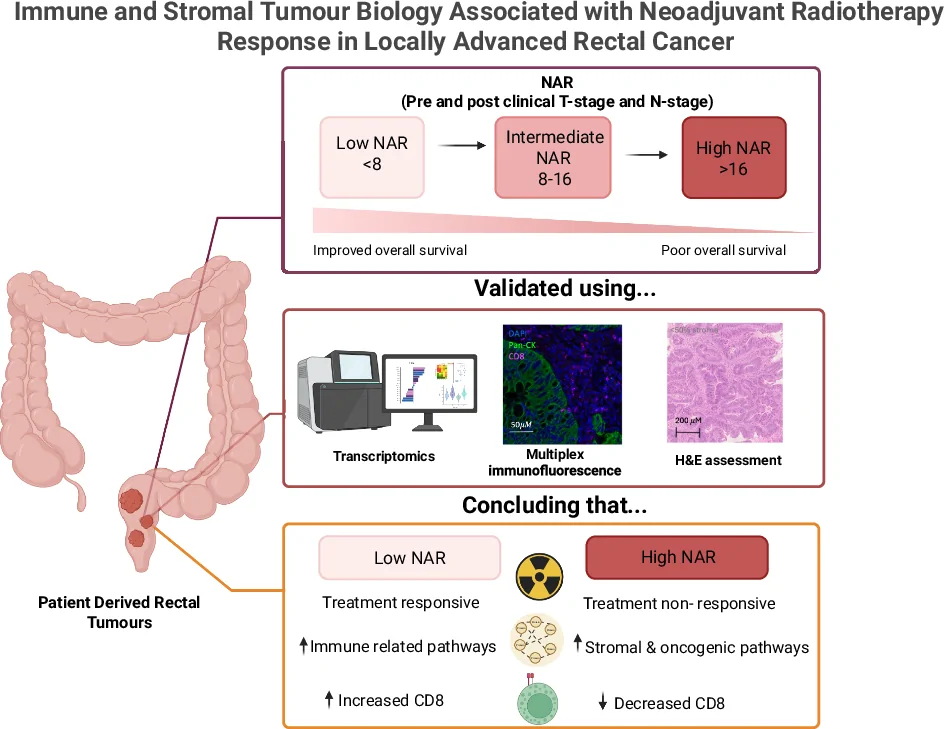

## Introduction

Despite widespread adoption of neoadjuvant radiation-based strategies and guidelines recommending their use in locally advanced rectal cancer (LARC) [1, 2], treatment responses are variable. Broadly speaking, after chemoradiotherapy, 20% of LARC patients achieve complete response, whereas approximately 10% exhibit little or no tumour regression [3, 4]. Response biomarkers are needed to optimise allocation of radiotherapy towards radiation-sensitive tumours whilst avoiding unnecessary, potentially morbid treatment in resistant disease.

The Neoadjuvant Rectal (NAR) score, initially proposed by George et al. 2015, incorporates T-stage downstaging (cT-ypT) and nodal status (ypN) from resection specimens (NAR = [5pN-3(cT-pT)+12]^2^/9.61). The focus on clinical downstaging intuitively links NAR to response, but it is also a potential surrogate endpoint for predicting longer-term outcomes [5]. When categorising patients based on NAR, NAR < 8 indicates good response associated with improved cancer outcomes, 8–16 represents intermediate response and > 16 indicates poor tumour response, associated with poorer long-term outcomes (local/distant recurrence and overall survival) [5]. This has been validated across a range of neoadjuvant regimens [6, 7]. Notably, the high NAR patient-group is clinically relevant as these patients have poorest long-term outcomes. Additionally, the NAR score has been employed as a primary trial endpoint [8].

There is convincing conceptual rationale for NAR as a short-term outcome, correlating with established long-term outcomes; overall-survival (OS) and disease-free-survival (DFS). It is unclear whether tumours with different NAR scores are biologically distinct. To date, an in-depth interrogation of the pre-treatment biology underpinning the high-NAR grouping has not been performed. This work aimed to utilise data from three cohorts with pre-treatment samples, (two with publicly available microarray data and one with immunostaining performed locally) to investigate biological differences between NAR categories with a particular focus on high and low-NAR tumours to contrast the more extreme clinical groups.

## Materials and Methods

### Clinical cohorts

Patients from the Beatson West of Scotland Cancer Centre (BWoSCC), Stratification in Colorectal Cancer (S:CORT) Grampian cohort and GSE87211 cohorts all had rectal adenocarcinomas. Informed consent from patients was obtained. All patients received neoadjuvant radiotherapy, followed by surgical resection. To calculate the NAR score, pre-treatment clinical T-stage determined by MRI, and a post-treatment T-stage and N-stage by pathologic staging were required.

The BWoSCC cohort consisted of *n* = 57 patients with pre-treatment biopsy tissue treated at the BWoSCC 2008–2016. 50 Gy of radiotherapy was delivered in 25 fractions with concurrent oral capecitabine or intravenous 5-fluorouracil-based chemotherapy. This study received ethical approval from the NHS Greater Glasgow and Clyde Research Ethics Committee (ref; 20/ES/0012). With informed consent.

The S:CORT consortium Grampian cohort comprised *n* = 193 rectal cancer patients who received neoadjuvant radiotherapy and has been described previously [9]. Patients received of 50 Gy of radiotherapy in 25 fractions or 25 Gy in 5 fractions. Additionally, some patients received capecitabine concurrently or 5-FU/oxaliplatin-based chemotherapy after radiotherapy.

GSE87211 comprised *n* = 200 rectal cancer patients enrolled in the CAO/ARO/AIO-944 or CAO/ARO/AIO-04 trial with transcriptomic microarray data available. Patients received 50.4 Gy of radiation in 28 fractions with concurrent 5-FU alone or in combination with oxaliplatin-based consolidation chemotherapy.

### Multiplex immunofluorescence

Multiplex immunofluorescence in the BWoSCC tissue was performed with Leica BOND RX (Leica Microsystems, Cambridge, UK) using Epredia’s (Runcorn, UK) UltraVision Quanto (UVQ) Detection System horse radish peroxidase (HRP) kit. In brief, slides we deparaffinised, then Bond™ Epitope Retrieval 2 (ER2; 97 °C for 30 min), Hydrogen Peroxide Block (10 min), Protein Block (5 min), primary antibody (10 min), Primary Antibody Amplifier Quanto (10 min), HRP Polymer Quanto (10 min) and Akoya (Marlborough, MA) Opal™ Fluorophore (10 min) sequentially. Antibody complexes were stripped with Bond™ ER2. Primary antibody and Opal™ Fluorophore pairs were applied in successive rounds in the order: (1) FOXP3(Abcam; Cambridge, UK; Ab20034) -Opal™480 (,(2) CD3-Opal™520, (3) CD8 (Dako Aligent, Santa Clara CA; M7103)-Opal™650, (4) Ki67-Opal™570, (5) Pan-cytokeratin (Thermofisher; Cheshire, UK; MS-343-P)-Opal™780. Spectral DAPI for 5 min (Akoya). Tissue was mounted in Thermofisher Prolong Diamond Mountant. Slides were scanned on a S60 Hamamatsu Nanozoomer (Hamamatsu, Hertfordshire,UK). Stained tissue was annotated and scored on QuPath [10]. Three representative areas totalling 1,080,000μM^2^ per biopsy were selected. Areas were manually annotated as tumour epithelium (pan-cytokeratin, positive DAPI positive), microenvironment (pan-cytokeratin negative, DAPI positive) or ignore (negative, abnormal morphology or artefact). CD8-positive cells were counted where staining surrounded a nucleus. Microsoft Excel was used to calculate cell densities by normalising CD8+ cells by total area of each compartment.

### Tumour stromal percentage (TSP)

TSP accessed using by scoring H&Es according to whether > 50%/ ≤50% of the tumour area was stroma.

### Tumour purity

Tumour purity was estimated using the ESTIMATE algorithm (Estimation of STromal and Immune cells in MAlignant Tumor tissues using Expression data), where stromal and immune scores were calculated from gene expression data and combined to generate the ESTIMATE score.

### Microarray

The S:CORT consortium retrieved archival formalin-fixed paraffin-embedded (FFPE) pre-treatment diagnostic biopsies which were processed for the Almac Diagnostics XCEL microarray as described previously [11]. Microarray data was checked by S:CORT consortium researchers then pre-processed where raw CEL files underwent robust multiarray average normalisation for Almac Diagnostic XCEL array with the *affy* package (v1.56.0). 193 samples had microarray data and sufficient clinical data for analysis.

Clinical and gene expression data for GSE87211 was downloaded from the gene expression omnibus. FFPE pre-treatment diagnostic biopsies were processed for Human 4344 K v2 array platform as described previously [12]. A total of 200 samples had microarray data and sufficient clinical data for analysis.

### Transcriptomic analyses

Differential gene expression between two groups were accessed using *limma* (v1.44.0). To compare expression of pre-defined pathway signatures between two groups, pairwise geneset enrichment analysis (GSEA) was performed. The mSigDB Hallmark signature set was accessed via the *msigdbr* (v7.5.1; [13]). A ranked gene list was prepared by ranking genes in the Limma output by t-statistic. The GSEA was performed on the ranked genelist using the fgsea() function from the package *fgsea* (v1.30.0; [14]). The package R *MCPcounter* (v1.2.0) was used to access the MCP (Microenvironment Cell Population) algorithm and calculate scores, which were visualised using the geom_boxplot() argument in the *ggplot2* R package (v3.5.1)

### Statistical analysis

Analysis of data was performed by generating Kaplan Meier curves using Survival version 3.7-0 in R Studio with R version 3.6.0 (RStudio, Boston, MA, USA). Univariate cox regression were performed and generated in SPSS version 29 (IBM SPSS, Chicago, IL, USA).

## Results

### Clinical cohort characteristics and replication of NAR as associated with long-term survival outcomes

Across S:CORT Grampian, GSE87211 and BWoSCC the majority of patients (63-70%) were male (Tables 1–3). Pre-treatment, across all three cohorts the majority of tumours were cT-stage 3 (74–91%) and cN+ (56–65%). Across these cohorts, 95% of patients received long course chemoradiotherapy, therefore all analysis of radiotherapy was combined. The NAR classifications were similar across the cohorts. Most were classified as intermediate by NAR score (44–56%). High-NAR patients were slightly more frequent in each cohort (27–32%) than low NAR 12–26%.

**Table 1:** Baseline clinicopathological characteristics for patients in the S:CORT Grampian cohort.

	Overall	Low NAR (< 8)	Intermediate NAR (8–16)	High NAR (> 16)	*p*-value X^2^
***n***	193	48	93	52	
**Age (%)**					0.892
< 55	32 (16.6)	8 (16.7)	14 (15.1)	10 (19.2)	
55–75	119 (61.7)	31 (64.6)	57 (61.3)	31 (59.6)	
> 75	42 (21.8)	9 (18.8)	22 (23.7)	11 (21.2)	
**Gender (%)**					0.698
Male	122 (63.2)	28 (58.3)	61 (65.6)	33 (63.5)	
Female	71 (36.8)	20 (41.7)	32 (34.4)	19 (36.5)	
**MRI cT (%)**					0.979
1	1 (0.5)	0 (0.0)	1 (1.1)	0 (0.0)	
2	28 (14.5)	7 (14.6)	13 (14.0)	8 (15.4)	
3	152 (78.8)	38 (79.2)	73 (78.5)	41 (78.8)	
4	12 (6.2)	3 (6.2)	6 (6.5)	3 (5.8)	
**MRI cN (%)**					0.007
0	86 (44.6)	29 (60.4)	42 (45.2)	15 (28.8)	
1	81 (42.0)	12 (25.0)	43 (46.2)	26 (50.0)	
2	26 (13.5)	7 (14.6)	8 (8.6)	11 (21.2)	
**ypT (%)**					< 0.001
0	33 (17.1)	32 (66.7)	1 (1.1)	0 (0.0)	
1	20 (10.4)	15 (31.2)	4 (4.3)	1 (1.9)	
2	45 (23.3)	1 (2.1)	37 (39.8)	7 (13.5)	
3	91 (47.2)	0 (0.0)	49 (52.7)	42 (80.8)	
4	4 (2.1)	0 (0.0)	2 (2.2)	2 (3.8)	
**ypN (%)**					< 0.001
0	143 (74.1)	46 (95.8)	93 (100.0)	4 (7.7)	
1	37 (19.2)	2 (4.2)	0 (0.0)	35 (67.3)	
2	13 (6.7)	0 (0.0)	0 (0.0)	13 (25.0)	
**pCR**					< 0.001
< pCR	162 (83.9)	18 (37.5)	92 (98.9)	52 (100)	
pCR	31 (16.1)	30 (62.5)	1 (1.1)	0 (0.0)	

*NAR* neoadjuvant rectal score, *pCR* pathological complete response. Statistical testing by chi squared (X^2^).

**Table 2:** Baseline clinicopathological characteristics for *n* = 200 patients in GSE87211.

	Overall	Low NAR (< 8)	Intermediate NAR (8–16)	High NAR (>16)	*p*-value X^2^
***n***	200	52	88	60	
**Age (%)**					0.483
<55	42 (21.0)	15 (28.8)	14 (15.9)	13 (21.7)	
55-75	135 (67.5)	31 (59.6)	64 (72.7)	40 (66.7)	
>75	23 (11.5)	6 (11.5)	10 (11.4)	7 (11.7)	
**Gender(%)**					0.159
Male	139 (69.5)	35 (67.3)	67 (76.1)	37 (61.7)	
Female	61 (30.5)	17 (32.7)	21 (23.9)	23 (38.3)	
**MRI cT (%)**					0.005
2	6 (3.0)	3 (5.8)	1 (1.1)	2 (3.3)	
3	182 (91.0)	48 (92.3)	85 (96.6)	49 (81.7)	
4	12 (6.0)	1 (1.9)	2 (2.3)	9 (15.0)	
**MRI cN (%)**					0.005
0	65 (32.5)	24 (46.2)	31 (35.2)	10 (16.7)	
1	131 (65.5)	28 (53.8)	56 (63.6)	47 (78.3)	
NA	4 (2.0)	0 (0.0)	1 (1.1)	3 (5.0)	
**ypT (%)**					<0.001
0	35 (17.5)	35 (67.3)	0 (0.0)	0 (0.0)	
1	21 (10.5)	17 (32.7)	2 (2.3)	2 (3.3)	
2	44 (22.0)	0 (0.0)	39 (44.3)	5 (8.3)	
3	88 (44.0)	0 (0.0)	46 (52.3)	42 (70.0)	
4	12 (6.0)	0 (0.0)	1 (1.1)	11 (18.3)	
**ypN (%)**					<0.001
0	143 (71.5)	52 (100.0)	86 (97.7)	5 (8.3)	
1	42 (21.0)	0 (0.0)	2 (2.3)	40 (66.7)	
2	15 (7.5)	0 (0.0)	0 (0.0)	15 (25.0)	
**pCR (%)**					<0.001
< pCR	165 (82.5)	17 (32.7)	88 (100.0)	60 (100.0)	
pCR	35 (17.5)	35 (67.3)	0 (0.0)	0 (0.0)	

NAR – neoadjuvant rectal score, pCR – pathological complete response. Statistical testing by chi squared (X2).

**Table 3:** Baseline clinicopathological characteristics for *n* = 52 patients BWoSCC.

	Overall	Low NAR (< 8)	Intermediate NAR (8–16)	High NAR (> 16)	*p*-value X^2^
***n***	52	6	29	17	
**Age (%)**					0.957
< 55	11 (21.2)	1 (16.7)	6 (20.7)	4 (23.5)	
55–75	36 (69.2)	4 (66.7)	20 (69.0)	12 (70.6)	
> 75	4 (7.7)	1 (16.7)	3 (13.0)	1 (5.9)	
**Gender (%)**					0.496
Male	33 (63.5)	5 (83.3)	18 (62.1)	10 (58.8)	
Female	19 (36.5)	1 (16.7)	11 (37.9)	7 (41.2)	
**MRI cT (%)**					0.430
2	3 (6.1)	0 (0.0)	2 (6.9)	1 (5.9)	
3	36 (73.5)	3 (0.5)	19 (34.5)	14 (82.3)	
4	10 (20.4)	2 (33.3)	7 (24.1)	1 (11.8)	
**MRI_cN (%)**					0.051
0	14 (26.9)	2 (33.3)	11 (37.9)	1 (5.9)	
1	27 (51.9)	2 (33.3)	16 (55.2)	9 (52.9)	
2	10 (19.2)	1 (16.7)	2 (6.9)	7 (41.2)	
**ypT (%)**					< 0.001
0	5 (9.6)	5 (83.3)	0 (0.0)	0 (0.0)	
1	3 (5.8)	0 (0.0)	3 (10.3)	0 (0.0)	
2	9 (17.3)	1 (16.7)	6 (20.7)	2 (11.8)	
3	35 (67.3)	0 (0.0)	20 (69.0)	15 (88.2)	
4	0 (0.0)	0 (0.0)	0 (0.0)	0 (0.0)	
**ypN (%)**					< 0.001
0	34 (65.4)	6 (100)	28 (96.6)	0 (0.0)	
1	12 (23.1)	0 (0.0)	1 (3.4)	11 (64.7)	
2	6 (11.5)	0 (0.0)	0 (0.0)	6 (35.3)	
**pCR (%)**					< 0.001
< pCR	47 (90.4)	1 (16.7)	29 (100)	17 (100)	
pCR	5 (9.6)	5 (83.3)	0 (0.0)	0 (0.0)	

*NAR* neoadjuvant rectal score, *pCR* pathological complete response. Statistical testing by chi squared (X^2^).

**Fig. 1:**
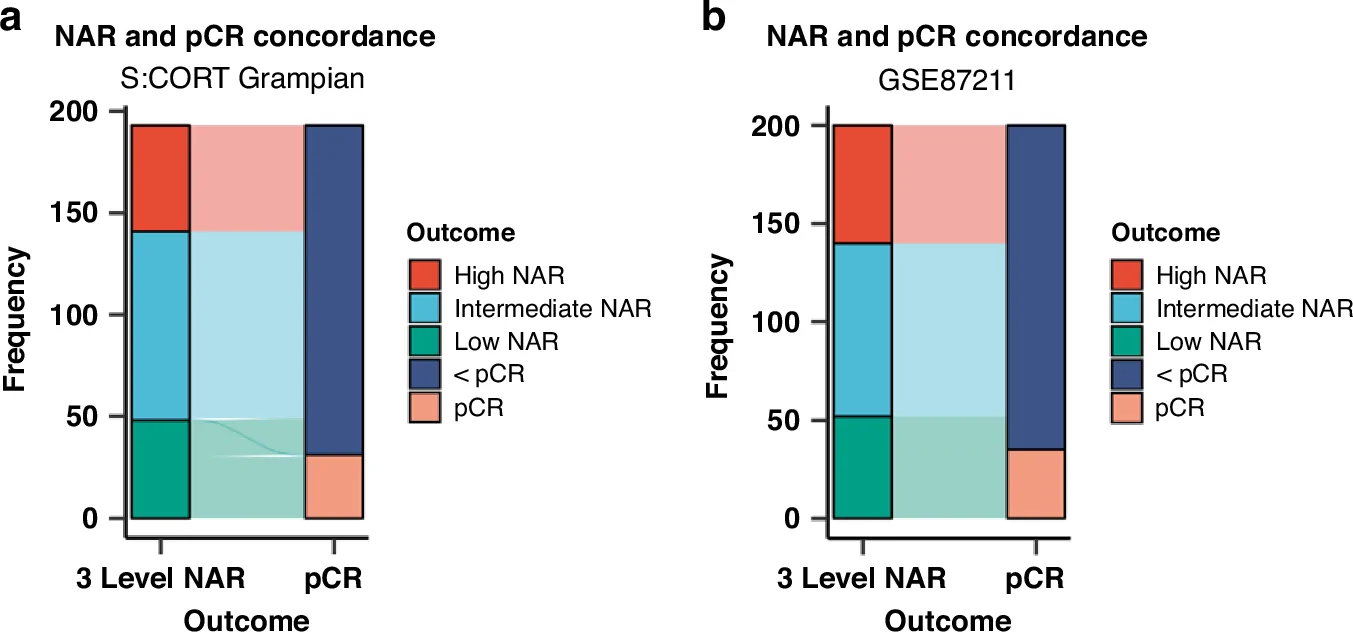
Sankey diagram showing the relationship between neoadjuvant rectal (NAR) categories and pathological complete response. NAR categories: low < 8, intermediate 8–16, high > 16. Pathological complete response categories: pCR and < pCR. **a** *n* = 193 S:CORT Grampian cohort, **b** *n* = 200 GSE87211. NAR neoadjuvant rectal score, pCR pathological complete response.

Figure 1 shows that when the NAR categories (low, intermediate and high) are compared to pathological complete response status (pCR or non-pCR), the incomplete response group comprises a range of NAR categories. The NAR score can therefore further stratify patients by the extent of non-response.

Assessment of Kaplan-Meir survival plots show a strong association between high NAR and shorter DFS and OS compared to low-NAR. On cox regression analysis for DFS a significant association with survival was observed for NAR in the S:CORT cohort (HR = 3.04, 95%CI 1.36–6.78, *p* = 0.007) and the GSE87211 cohort (HR = 2.41 95%CI 1.69–3.42, *p* < 0.01). This observation was replicated in all three cohorts (supplementary fig. 1)

### Enrichment for hallmark epithelial-to-mesenchymal transition and key colorectal cancer canonical driver pathways in high-NAR tumours

**Fig. 2:**
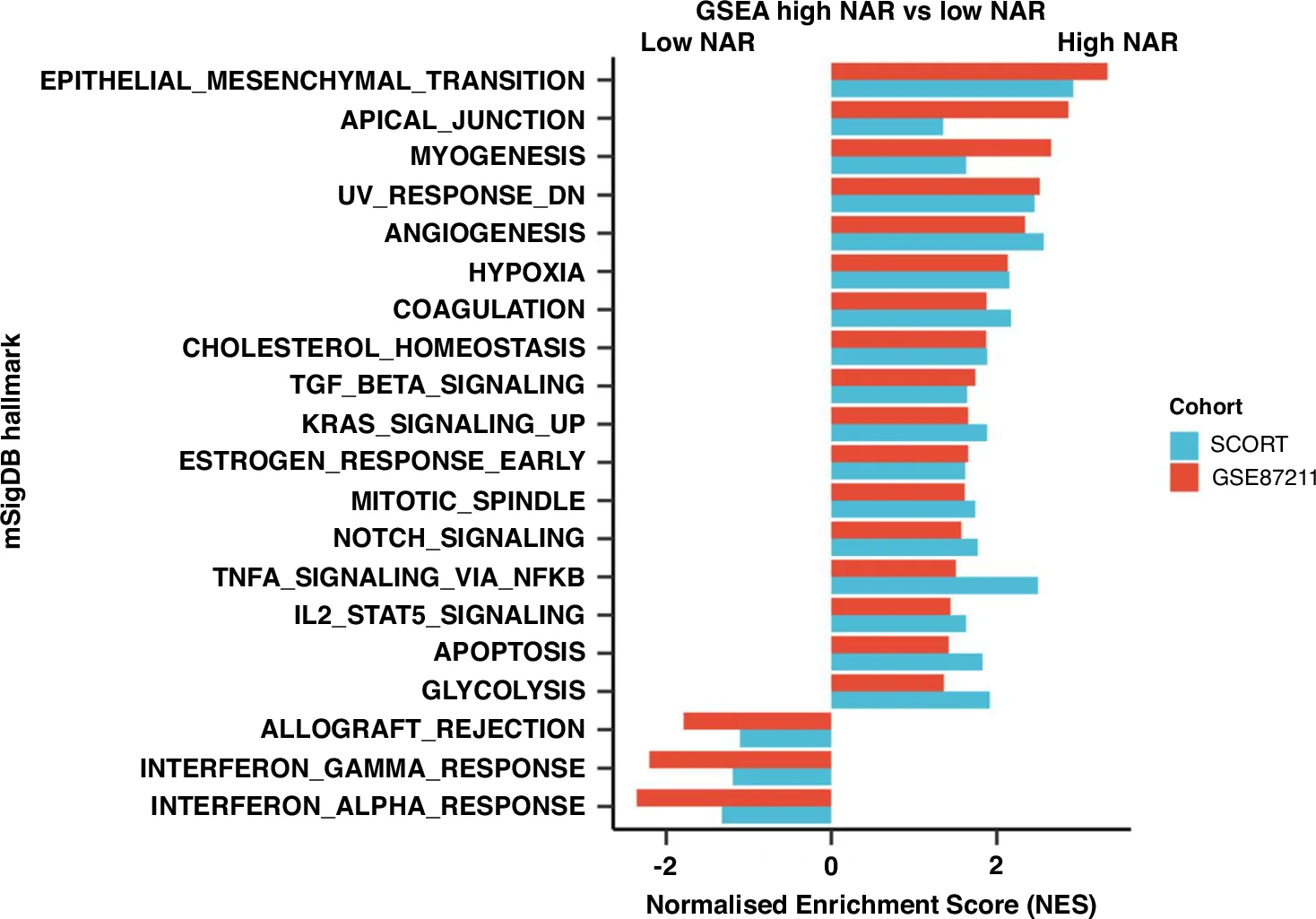
Pairwise gene set enrichment analysis comparing high NAR and low NAR tumours. Barplot of the normalised enrichment scores (NES) for pathways that are positively ( + ve NES) or negatively enriched (−ve NES) for high NAR tumours compared to low NAR (p-adjust < 0.25).

The enrichment results from both cohorts were compared, identifying overlap *n* = 17 signatures enriched in high-NAR tumours (padj < 0.25, NES > 1.5) and *n* = 3 signatures enriched in low-NAR tumours (padj < 0.25, NES < −1.5) (Fig. 2). Notably, ‘Hallmark Epithelial-to-Mesenchymal Transition’ (EMT); key canonical drivers of colorectal cancer tumorigenesis ‘Hallmark Wnt Beta Catenin Signaling’, ‘Hallmark TGF-Beta Signaling’ and ‘Hallmark P53 Pathway’; and inflammation-related pathways ‘Hallmark Inflammatory Response’ and ‘Hallmark IL6-JAK STAT Signalling’ were all enriched in high-NAR tumours. All three pathways enriched in low-NAR tumours were annotated as ‘Immune’: ‘Hallmark Allograft Rejection’, ‘Hallmark Interferon-Gamma Response’ and ‘Hallmark Interferon-Alpha Response’[13] (Fig. 2). In a recent study, these three pathways were identified as enriched in purified leukocytes compared to stromal and epithelial cells [15]. Conversely, the genes in signatures enriched for high-NAR tumours such as ‘Hallmark Epithelial-to-Mesenchymal Transition’, ‘Hallmark Wnt Beta Catenin Signaling’ and ‘Hallmark TGF-Beta Signaling’, ‘Hallmark Apical Junction’ and ‘Hallmark Myogenesis’ are enriched in stromal populations [15]. GSEA plots of these findings as shown in Fig. 2 in the supplementary data confirm these findings with stricter thresholds.

### Higher immune infiltration in low-NAR tumours

**Fig. 3:**
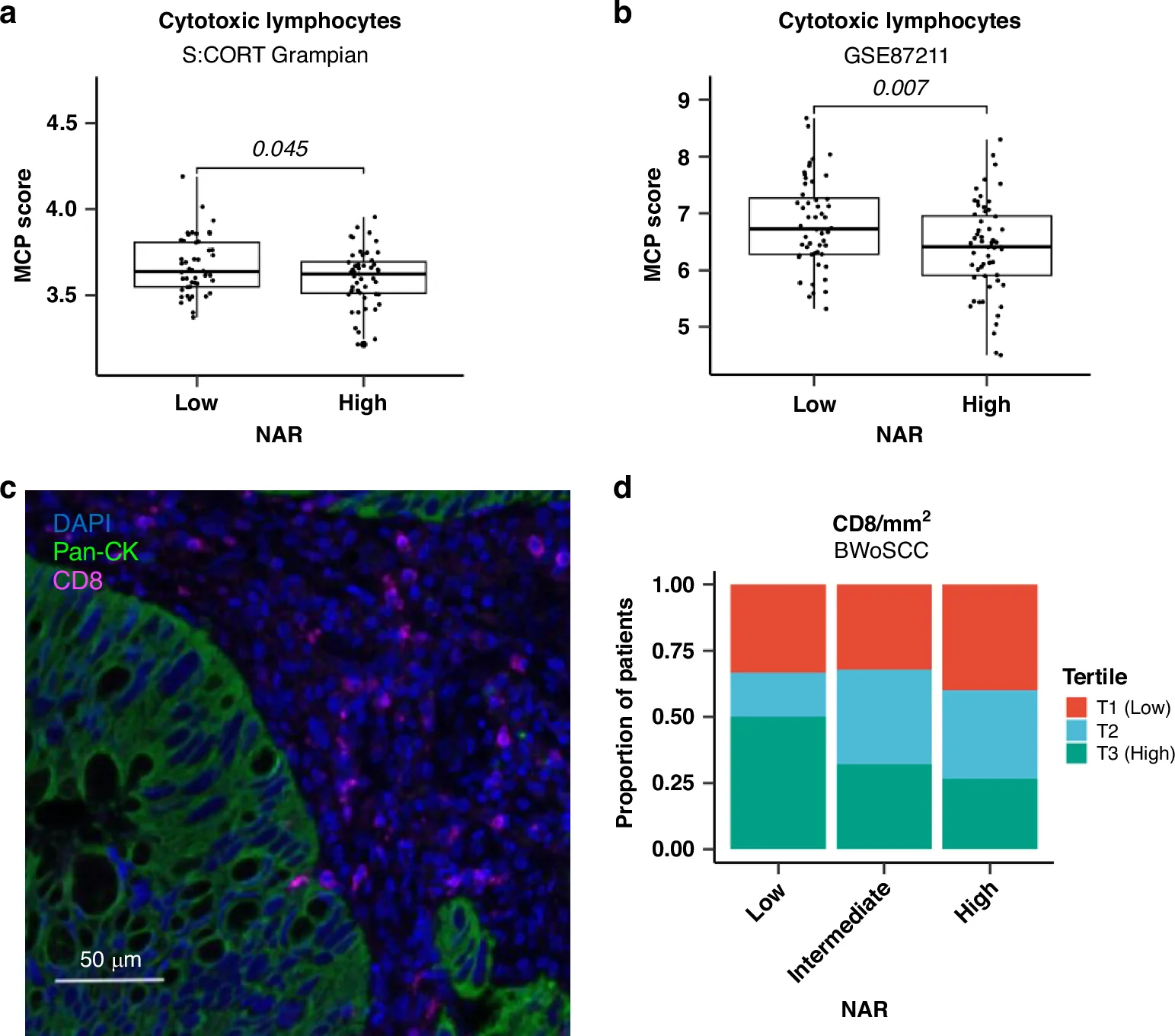
Differential MCP T cell populations between High NAR and Low NAR tumours. Microenvironment cell population scores for (**a**, **b**) Cytotoxic Lymphocytes, (**c**) CD8, pan-cytokeratin and DAPI representative image (**d**) stacked bar plot of the distribution of high, medium and low tertiles of CD8 density scores across the low (*n* = 6), high (*n* = 17) and intermediate (*n* = 29) NAR categories.

The GSEA revealed enrichment for stromal-associated pathways in high-NAR tumours and immune-associated pathways in low-NAR tumours. Next, we assessed whether transcriptional differences between high and low-NAR groups were associated with differing abundances of microenvironment lineages. The MCP counter found significantly higher cytotoxic lymphocytes in low vs high-NAR tumours in both S:CORT (*p* = 0.045) and GSE87211 (*p* = 0.007) (Fig. 3a, b). However, the interquartile ranges of the MCP cytotoxic lymphocytes scores in high and low-NAR groups overlapped and the differences between the groups were insufficient for stratification. Within the Glasgow BWoSCC cohort (*n* = 52), analysis using multiplex immunofluorescence demonstrated a higher proportion of CD8+ densities in the upper tertile (50%) in the low-NAR tumours compared with high-NAR tumours (25%) (Fig. 3c, d) although this difference was not significant (p=ns).

### Larger transcriptomic stromal cell populations in high-NAR tumours

**Fig. 4:**
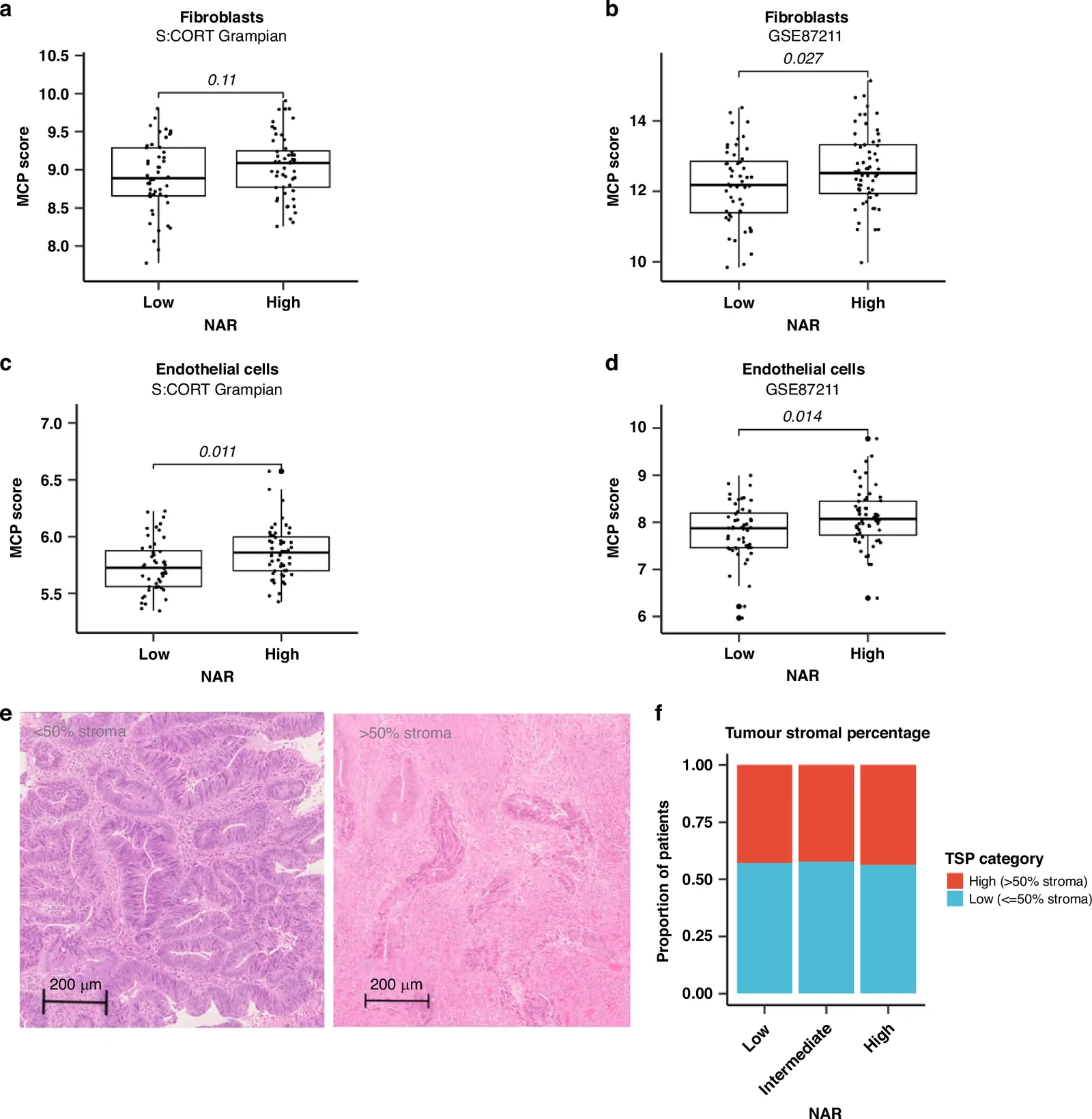
Microenvironment cell population (MCP) scores for stromal populations in Low NAR and High NAR samples. **a**, **b** Fibroblasts. **c**, **d** Endothelial cells. Statistical testing by T-test. Tumour stromal percentage with a 50% cut off was used. **e** Example of low stromal volume ( < 50%) and high stromal volume ( > 50% tumour). **f** Stacked bar plot showing the proportion of high (*n* = 17), intermediate (*n* = 29) and low (*n* = 7) NAR tumours which were high stroma of low stroma

MCP identified higher stromal populations in high vs low-NAR tumours in both S:CORT and GSE87211 (Fig. 4a–d). MCP fibroblasts scores were significantly higher in high-NAR tumours in GSE87211 (*p* = 0.027) but not S:CORT Grampian (*p* = 0.11). MCP endothelial cells were significantly higher in high-NAR tumours in S:CORT Grampian (p = 0.011) and GSE87211 (*p* = 0.014). To investigate whether the association of MCP immune and stromal cell populations with NAR just reflected tumour purity, a tumour stromal percentage (TSP) assessment was performed in the BWoSCC cohort to validate whether a greater stomal volume was associated with poor response to neoadjuvant treatment. The proportion of high stroma tumours was similar across NAR categories (Fig. 4f), thus tumour purity assessed by TSP in this cohort was not associated with NAR. *In lieu* of a TSP assessment for the publicly available datasets and ESTIMATE score was calculated to infer tumour purity from the transcriptomic data, however we did not identify a significant difference between NAR categories (Suppl. Fig. 3).

### Combination of stromal and immune populations to prediction NAR score

There was no association between MCP fibroblasts and cytotoxic lymphocytes in either S:CORT Grampian (*r* = −0.03) nor GSE87211 (*r* = 0.01; Suppl. Fig. 4A, B). Thus, whilst these populations were associated with NAR category separately, the MCP stomal scores and cytotoxic lymphocyte scores are independent from each other. The ten pathways with the highest NES in GSE87211 were highly correlated with MCP stromal populations and monocytic lineage (Suppl. fig. 4C, D) suggesting that a stromal-inflammatory biology may be associated with the pathways enriched for the high-NAR patients.

**Fig. 5:**
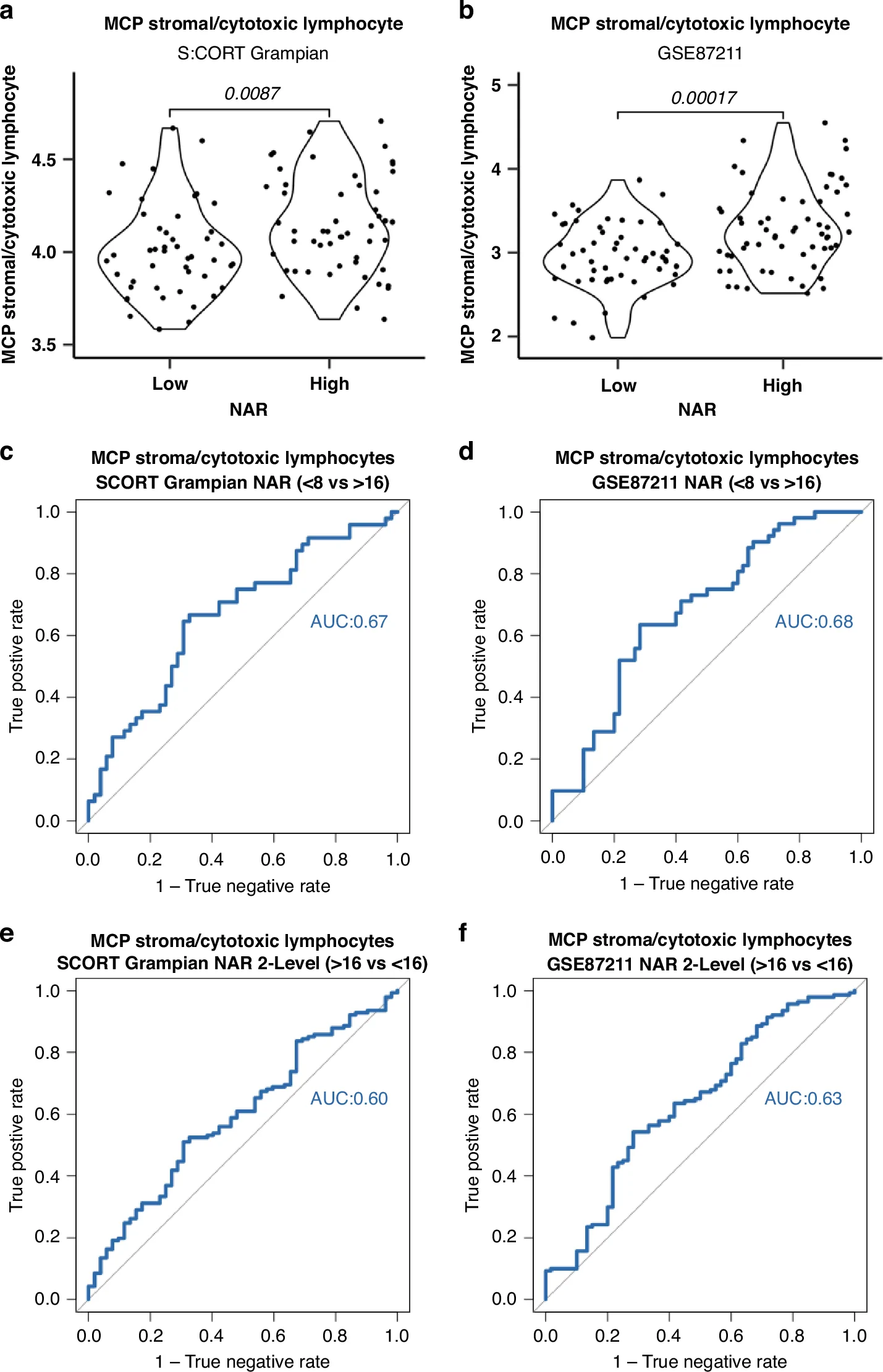
Combination of MCP scores to improve predictive ability of NAR Stromal/Cytotoxic Lymphocyte score ((MCP Fibroblast + MCP Endothelial Cell)/MCP cytotoxic lymphocyte). **a**, **b** Violin plot comparison of Stromal/Cytotoxic Lymphocyte score in High NAR and Low NAR patient tumours tested by T-test. **c**, **d** ROC curves for MCP stromal/cytotoxic lymphocyte score in cohorts of High NAR and Low NAR score patients only. **e**, **f** and across all patients with a threshold of ( > 16 = ‘High’, and < 16 = ‘Low’).

MCP scores for stromal and immune populations were combined to assess combined prediction of NAR score. MCP fibroblasts, endothelial cells and cytotoxic lymphocytes were used due to individual associations in both cohorts. The MCP fibroblasts score was added to the MCP endothelial cells and divided by MCP cytotoxic lymphocytes. The combined score was significantly higher in ‘High’ NAR tumours in S:CORT (*p* = 0.0087) and GSE87211 (*p* = 0.00017), indicating that high-NAR tumours had a higher ratio of stromal to cytotoxic cells pre-treatment (Fig. 5a, b). Using this score to stratify the high and low-NAR tumours the AUC was 0.67–0.68 (Fig. 5c, d). When the combined score was applied to the full cohort of patients to assess its ability to stratify around a threshold of NAR = 16, the AUC was 0.60–0.63 across the cohorts, representing, a modest predictive ability only (Fig. 5e, f).

## Discussion

This study used both microarray transcriptomic data and immunofluorescence in pre-treatment samples reporting that the least responsive, high-NAR tumours are enriched for key colorectal cancer driver pathways and stromal cell populations, whilst responsive low-NAR tumours are enriched for immune-related pathways, a finding validated by presence of higher cytotoxic T-cell (CD8 + ) infiltrates. Due to the inherent nature of these publicly available datasets different microarray platforms were utilised. However, considering this limitation the results highlight pre-treatment biological differences are reproduced across three independent cohorts, and importantly, consistent differences in differentially expressed signatures were observed despite the use of two distinct analytical platforms. This underscores the value of using gene signatures rather than individual genes to capture biological patterns across different platforms. Although the BWoSCC cohort is limited by its small number, with only 6 low NAR cases, the results still exhibited similar trend. However, consistent with other studies, these associations fail to demonstrate absolute divergence between responders and non-responders [11, 16].

The degree of immunogenicity has previously been implicated as an important factor in LARC radiotherapy response. Previously, transcriptomics showed that the ‘hot’ immune signature in the tumour before treatment is associated with an improved response [17]. Domingo et al. reported that the immunogenic CMS1 subtype was positively associated with pathological complete response [11]. Furthermore, El Sissy et al. observed pre-treatment CD3+ and CD8+ densities in biopsies relate to improved radiotherapy response[16, 18]. Importantly, recent trials within the immune ‘hot’ mismatch-repair deficient subset of LARC demonstrated promising results with immune targeting. Cercek et al. reported Dostarlimab alone (anti-PD-1) was associated with clinical complete response in all 12 patients [19].

The association of EMT and stromal gene expression with poor outcomes has previously been reported. Wilkins et al. 2021 reported higher pre-treatment ESTIMATE stromal score was associated with a poorer response [20]. Additionally, fibroblast protein, decorin is associated with prognosis in LARC [21]. Hallmark EMT is not intended to measure stroma but has become known as a ‘stromal’ gene signature [15]. In numerous independent cohorts Hallmark EMT was enriched in poor responding tumours before treatment [20, 21]. Furthermore, Wilkins et al. 2021 reported an enrichment for M2 (CD133 + ) macrophages and chronic inflammation in poor responding samples [20]. Domingo et al. similarly reached the conclusion that the inflammatory-stromal CMS4 subtype was associated with incomplete response [11]. However, after irradiation interleukin-1a has been demonstrated to polarise cancer-associated-fibroblasts toward a cancer-promoting inflammatory phenotype interleukin-1a [21]. With the stromal-activation changeable over the course of treatment, predictive ability of it as a baseline marker may be limited.

To-date, gene expression analyses have not delivered reproducible genes nor signatures predicting response to RT in LARC. A previous review of genes associated with response to radiotherapy reported no overlap between studies [22]. However, with the data presented in this paper, and other recent studies [11, 20, 21], a convergence on a stromal-inflammatory biology prior to treatment associating with poor response has begun to emerge. Domingo et al. completed a sophisticated integration of these signals with a locked machine learning model which achieved 74–89% AUC for pathological complete response depending on the batch correction approach [11].

Tumour biopsy heterogeneity presents a challenge in biomarker development. Biopsies have demonstrated concordance with the primary tumour core in the immunohistochemistry [6]. However, it has been recognised that samples taken from different regions of the same tumour can exhibit different transcriptomic profiles [23]. The differential proportions of stromal, immune and epithelial populations in the tumour core and periphery will influence the expression of certain gene signatures, making their interpretation challenging. This is especially challenging in the context of bulk transcriptomics, where it is not possible to determine the cell-type source of gene expression [15]. Indeed, within the data presented here there is an association between enriched pathways and microenvironment populations. In the future, spatial transcriptomic approaches or additional multiplex immunofluorescence panels should allow us to determine which compartment is driving the expression or enrichment of genes or processes. Biopsies only represent a small part of a heterogeneous tumour, resulting in much of the tumour biology being unseen. This presents a challenge for biomarker research as biopsies may not be representative of the biology driving the tumour.

Previously, studies have focussed on using pathological complete response as an endpoint for response to chemoradiotherapy, selecting the small subgroup of best responders, comparing these to incomplete response. The latter patient group is a heterogeneous mixture of poor, intermediate and near-complete responses. The NAR method allows selection of the best and worst responders, thus representing two clinically very different groups for comparison. Developing robust and reliable predictors of resistance to radiotherapy (high-NAR > 16) would have profound implications for disease stratification. Clinicians could avoid prolonged, potentially toxic treatments in patients unlikely to benefit. Although some biological differences between NAR categories have been identified in the current study, given the modest AUC alternative methods of integrating signatures, additional external validation work is required and the consideration of further contributing biologies should be considered. The complex interplay between rectal tumour epithelium and constituent cells within the microenvironment is likely to change considerably after radiation is initiated [24]. Moving beyond interrogation of baseline pre-treatment samples is therefore a priority.

## Conclusion

Using transcriptomic and immunohistochemical analyses across multiple cohorts we report low-NAR (responsive) tumours are enriched for immune-related pathways and higher CD8 + T cell infiltrates, while high-NAR (non-responsive) tumours are enriched for stromal and oncogenic pathways. However, these findings highlight the complexity of radiotherapy response, as pre-treatment microenvironment infiltration alone does not fully discriminate responders and non-responders, and a high degree of heterogeneity remains.

## Supplementary information

Supplementary Material

## Data Availability

Data from the BWoSCC is available upon request from the authors. The S:CORT Grampian dataset is publicly available and can be accessed from the primary transcriptomic publication for the S:CORT consortium https://doi.org/10.1016/j.ebiom.2024.105228. Data for GSE87211 is publically available on the Gene Expression Omnibus: https://www.ncbi.nlm.nih.gov/pubmed/29119627.

## Code availability

All code available by contacting the authors.
