# Supplementary material for: Immune and stromal tumour biology associated with neoadjuvant radiotherapy response in locally advanced rectal cancer
Source: Br J Cancer. 2026 Aug 18;135(10):1705–14. doi: 10.1038/s41416-026-03575-y (PMC13739902; doi:10.1038/s41416-026-03575-y)
Supplement: Supplementary file 1 [file 41416_2026_3575_MOESM1_ESM.pdf]

Supplementary figures

A

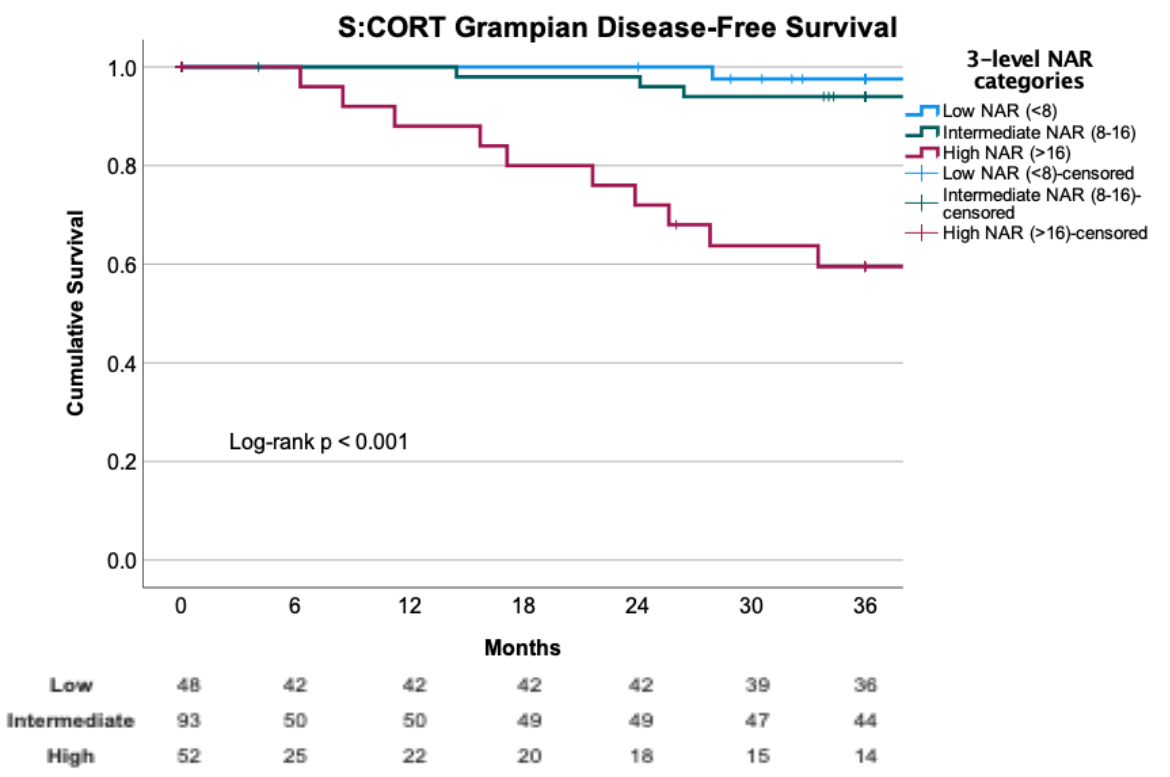

B

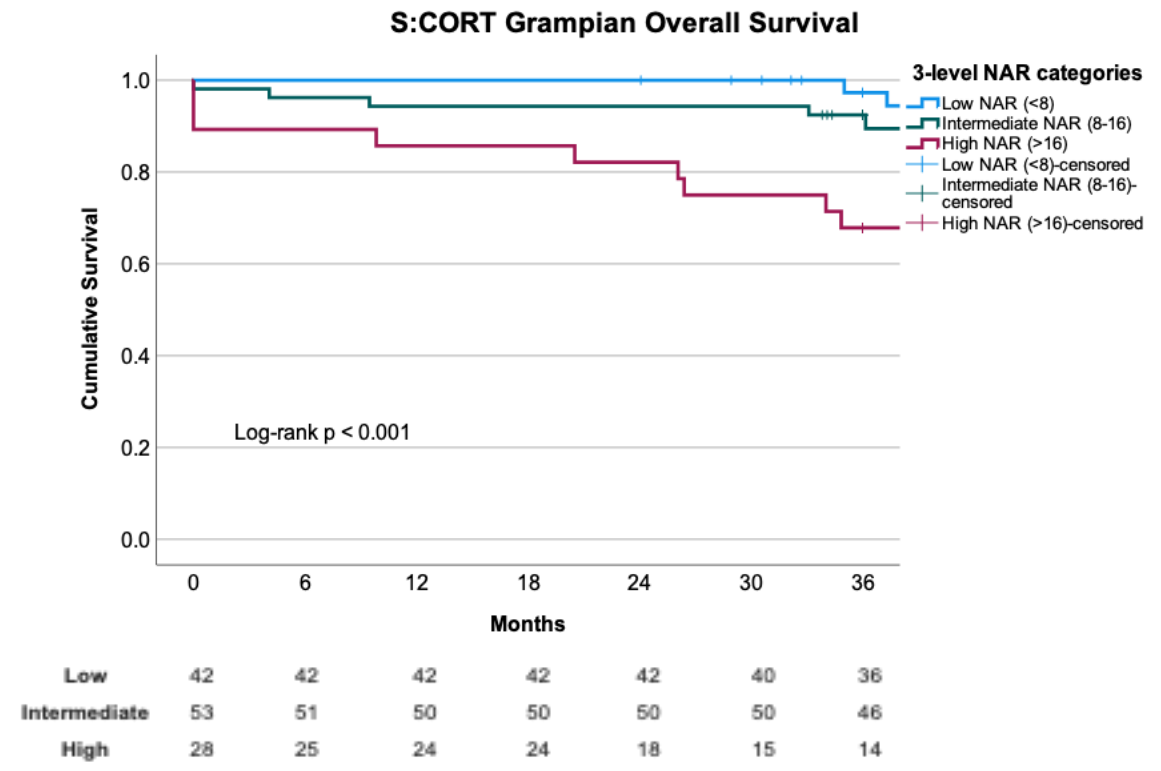

C

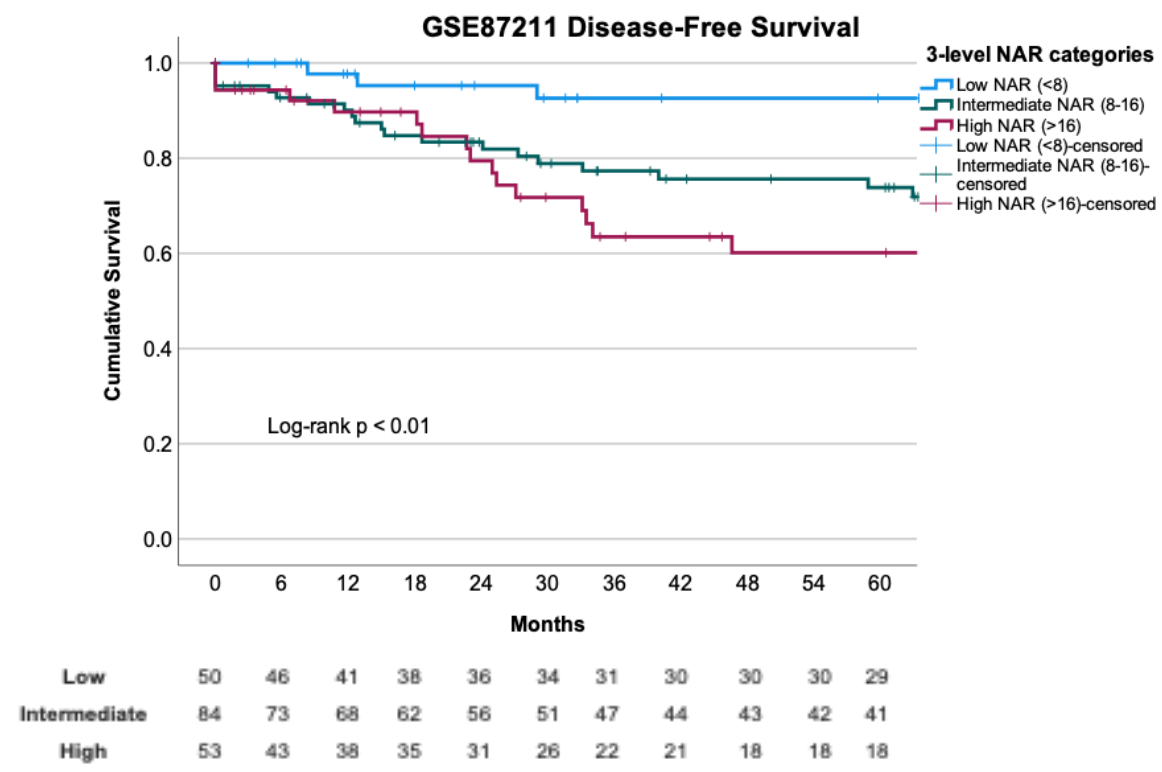

D

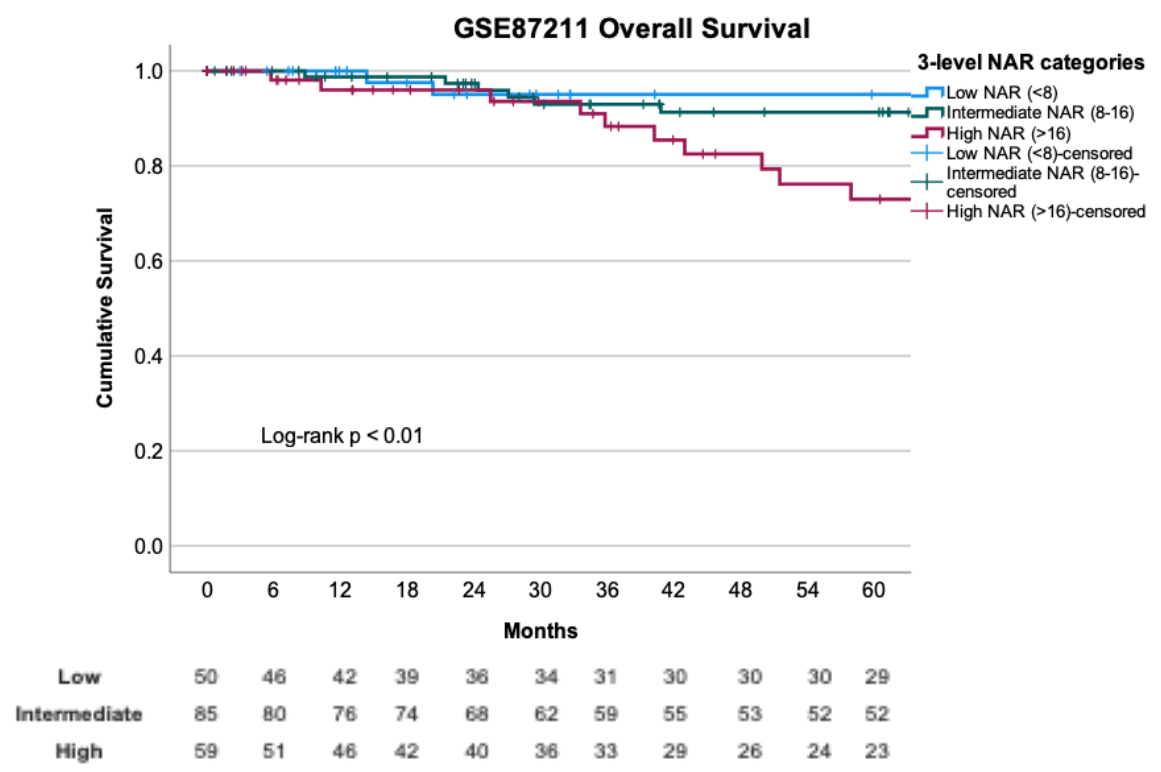

E

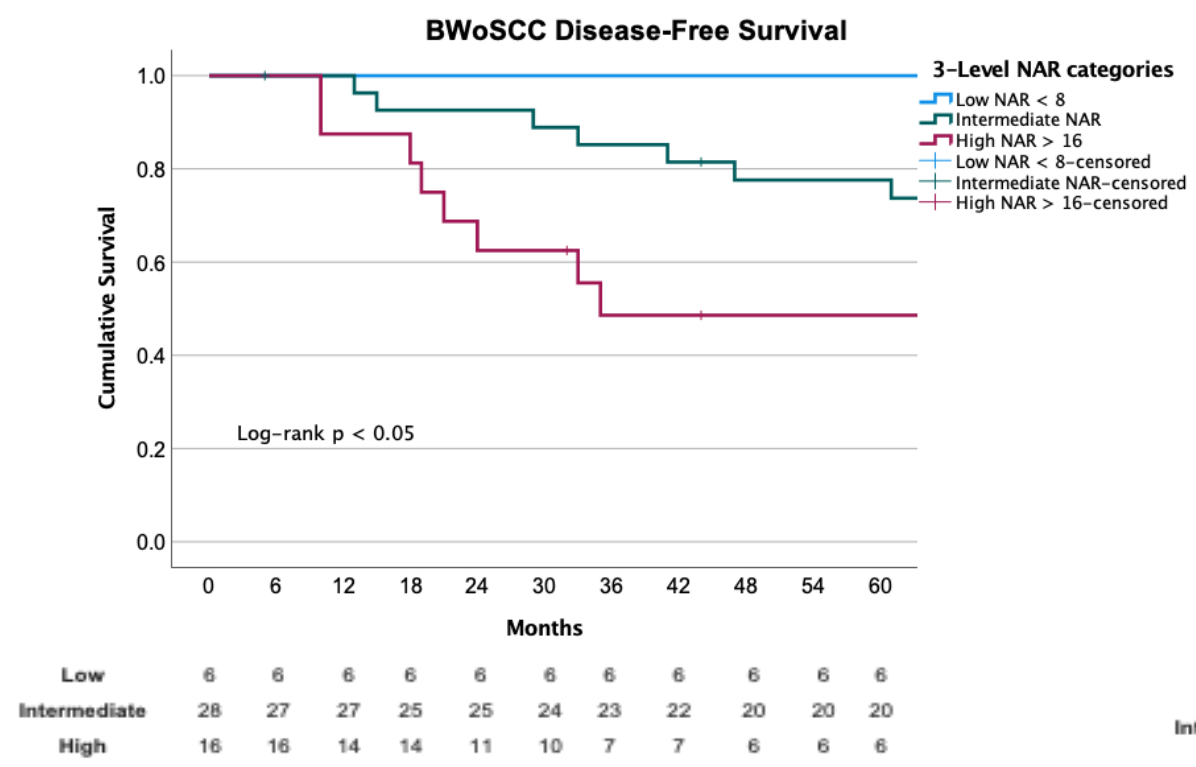

F

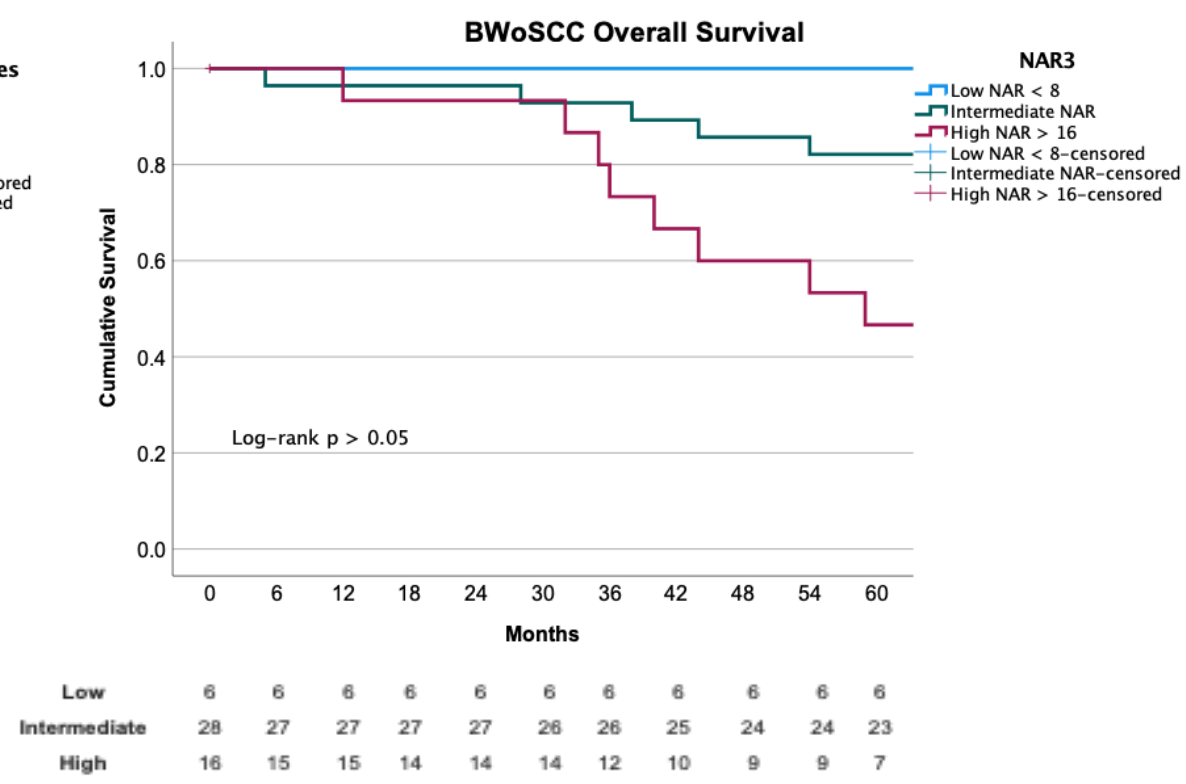

Supplementary figure 1 Kaplan-Meier plots for NAR categories, disease-free survival and overall survival. Statistical analysis using log-rank test. In (A,B) S:CORT Grampian cohort for 36 months (n=193)(C,D) GSE87211 for 60 months (n=187) (E,F) Beatson West of Scotland Cancer Cohort for 60 months (n=50).

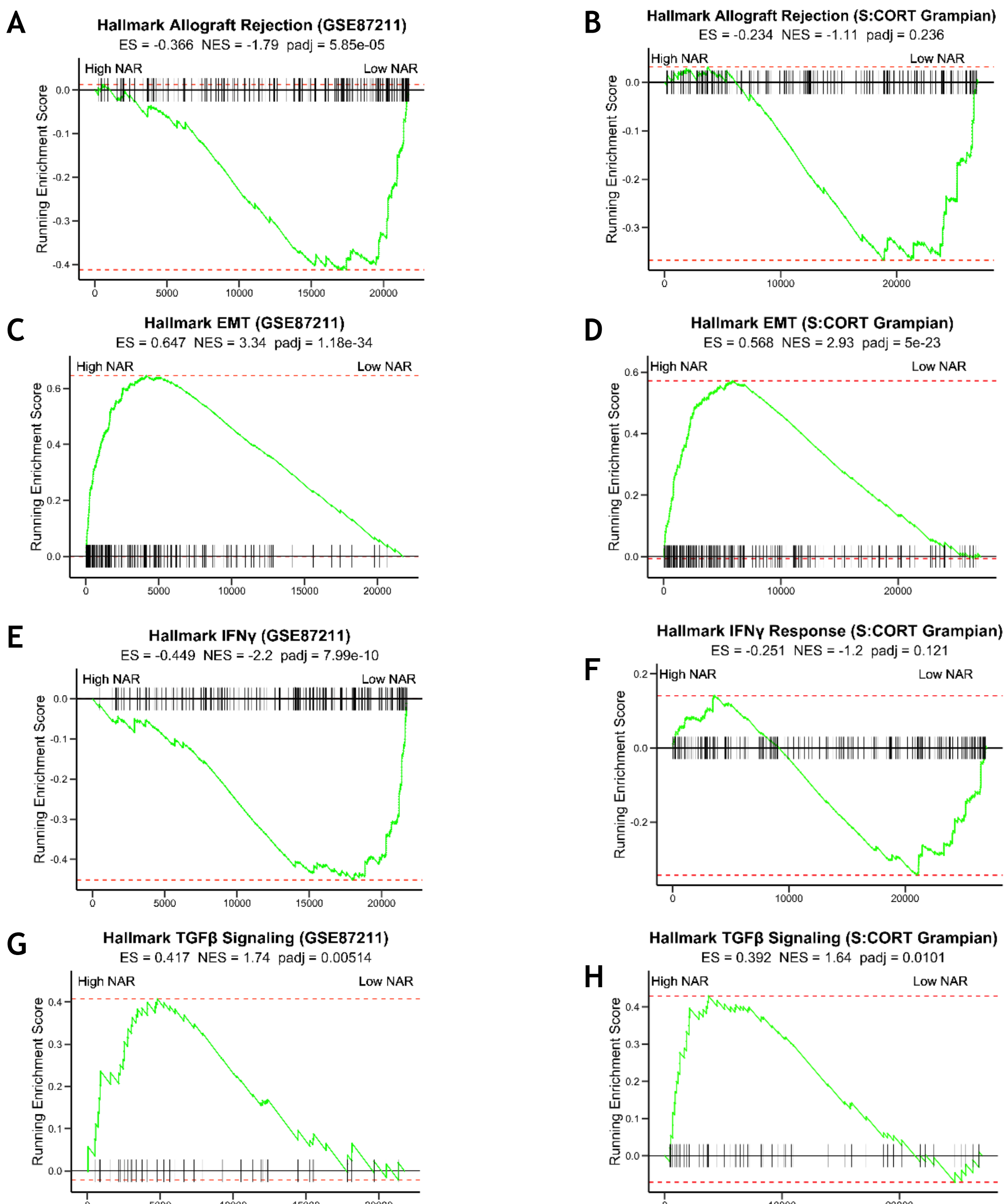

Supplementary figure 2 Pairwise gene set enrichment analysis comparing high NAR and low NAR tumours. GSEA enrichment plot of the normalised enrichment scores (NES) for pathways that are positively (+ve NES) or negatively enriched (-ve NES) for high NAR tumours compared to low NAR. A,C,E,G GES87211 cohort Hallmarks Allograft (p-adjust <5.85e-05), EMT (p-adjust <1.8), IFNγ (p-adjust <7.99e-010), TGFβ (p-adjust <0.00514) respectively and B,D,F,H S:CORT cohort Hallmarks Allograft (p-adjust <0.236), EMT (p-adjust <5e-23), IFNγ (p-adjust <0.121), TGFβ (p-adjust <0.0101) respectively.

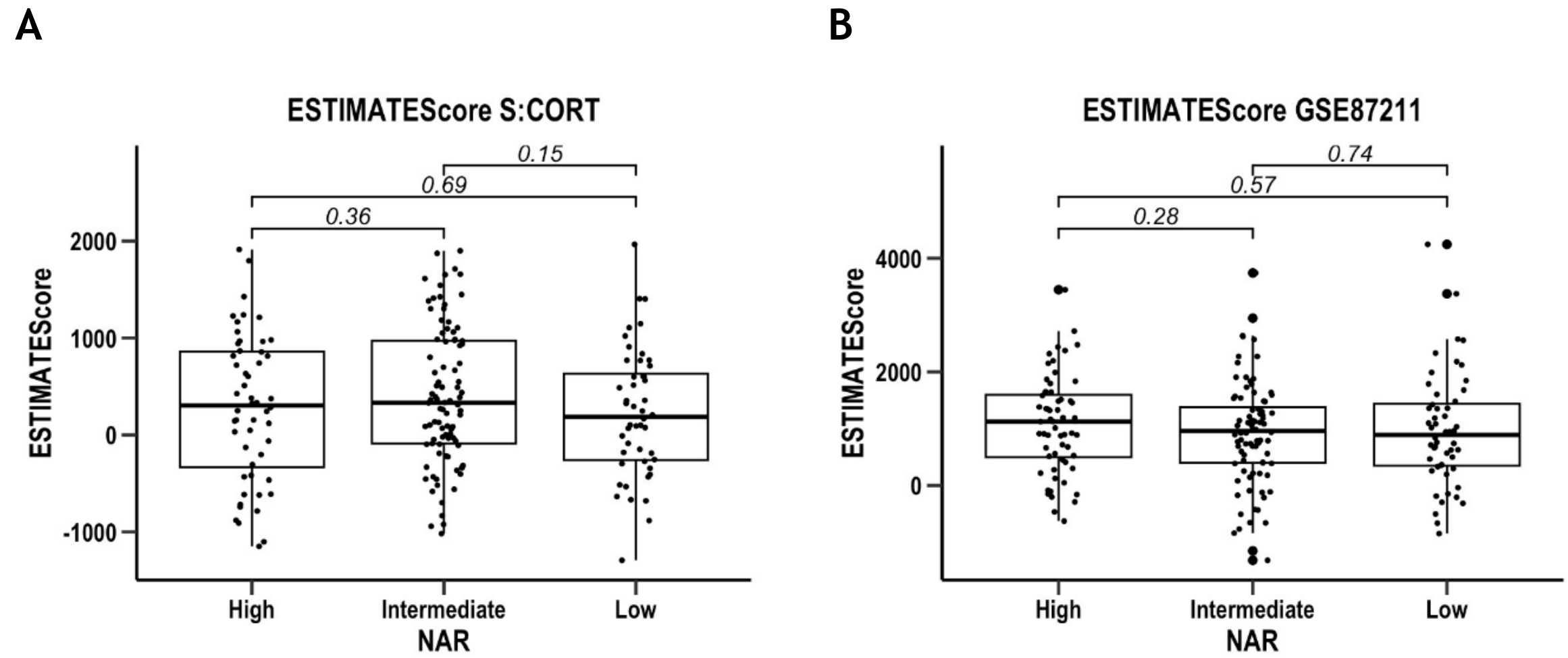

Supplementary figure 3: EstimateScores for each NAR category. (A) S:CORT Grampian cohort (n=193). (B) GSE87211 (n=200).

A

S:CORT Grampian

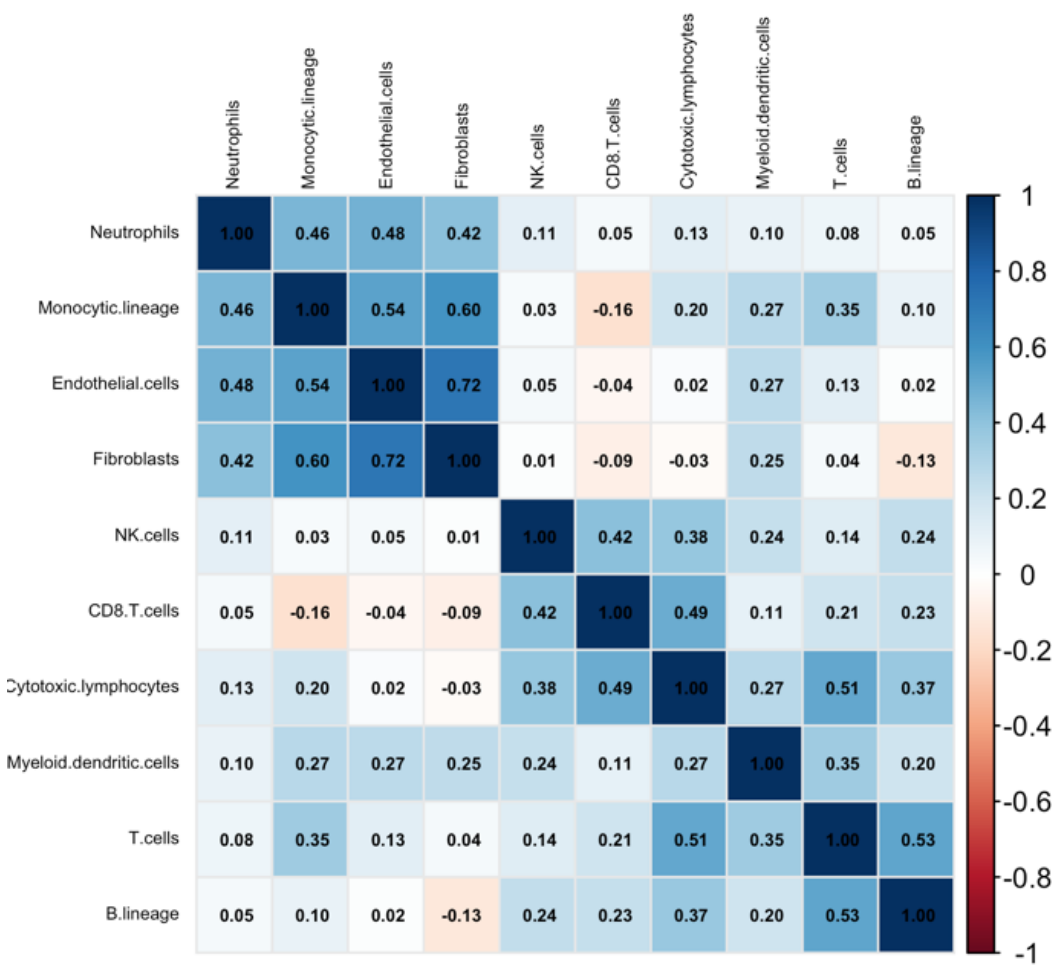

B

GSE87211

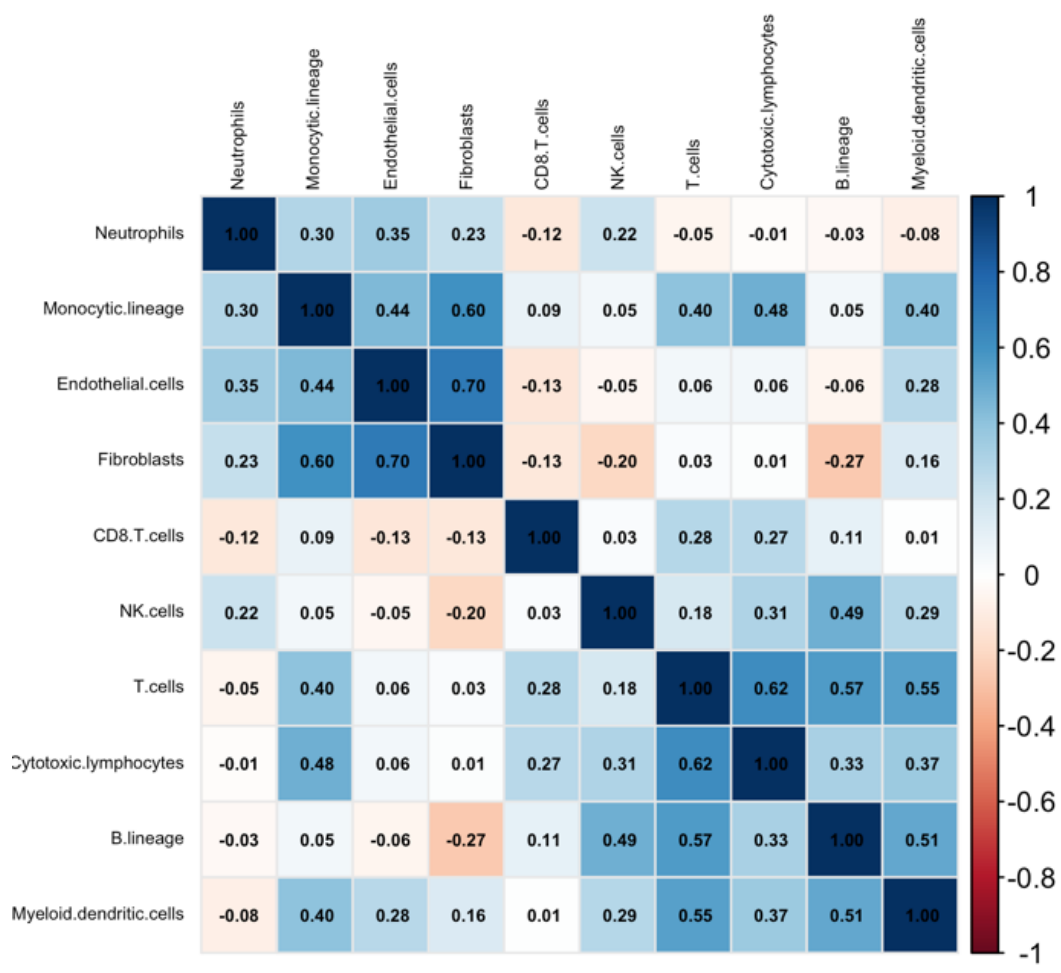

C

S:CORT Grampian

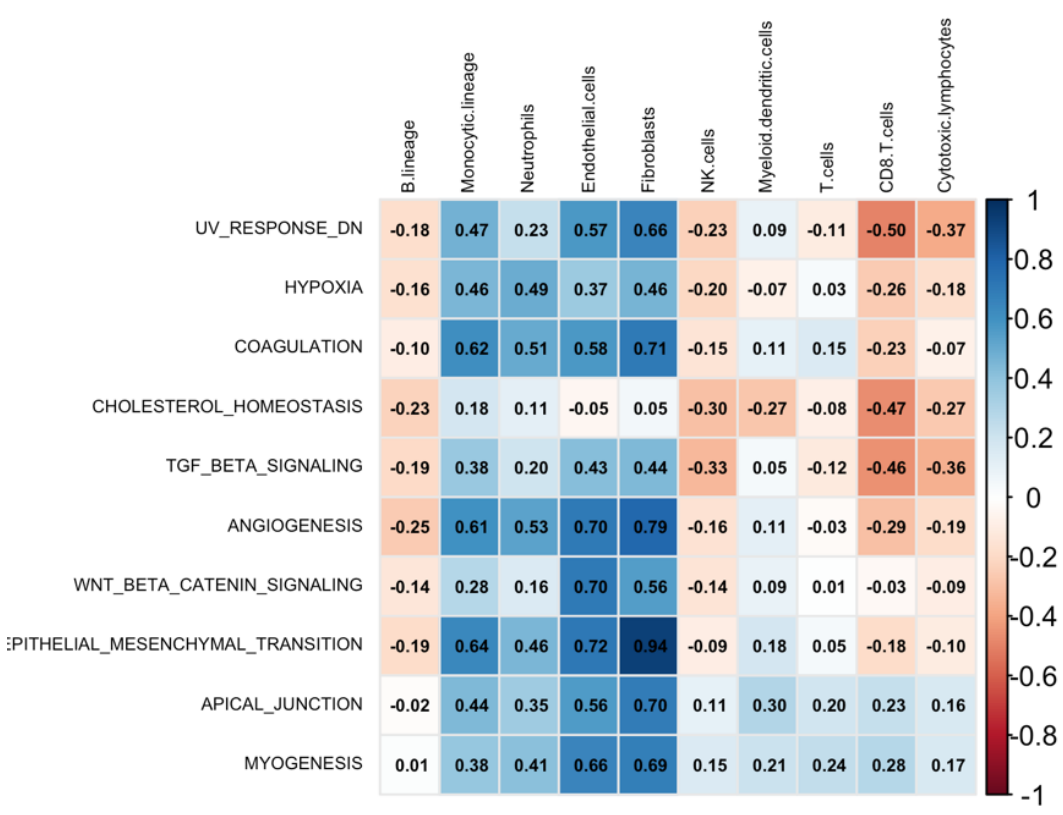

D

GSE87211

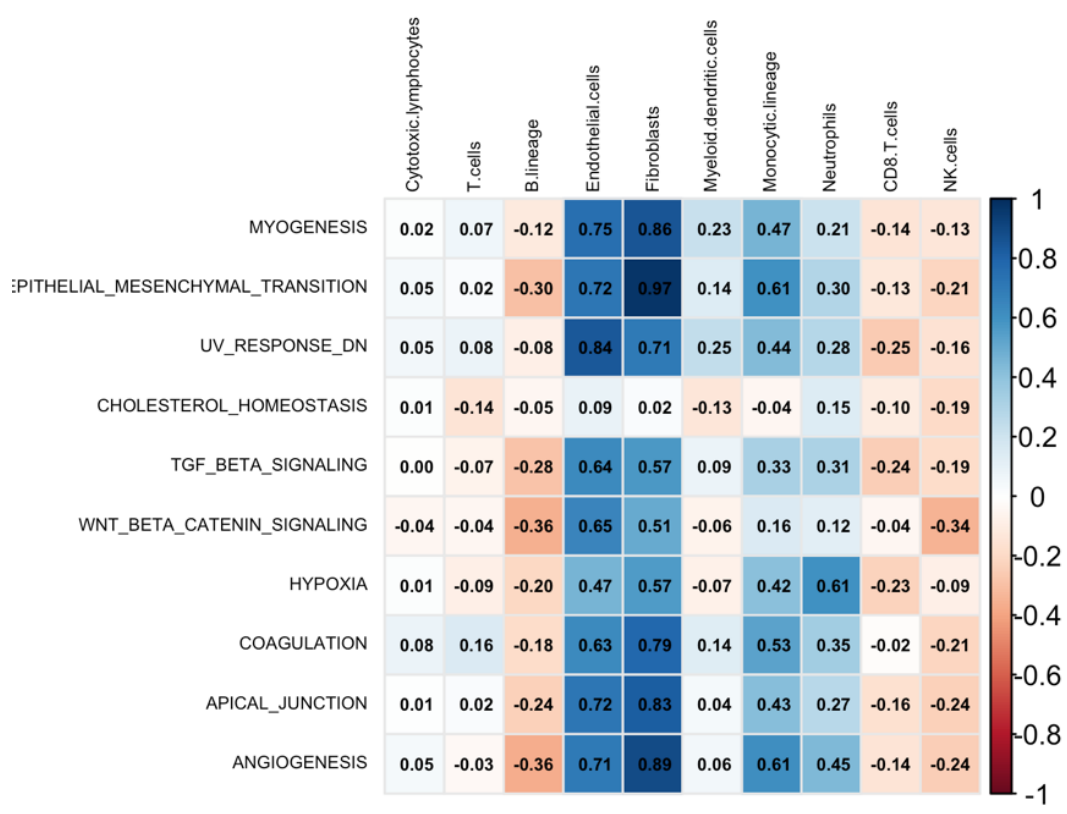

Supplementary figure 4: Correlation matrices for the association of microenvironment cell population (MCP) counter scores with each other (A) S:CORT Grampian cohort (n=193). (B) GSE87211 (n=200). Pearson correlation co-efficient plotted. Correlation matrices for the association of microenvironment cell population counter scores with single sample geneset enrichment scores for the top 10 pathways enriched for high neoadjuvant rectal score (NAR) tumours. (C) S:CORT Grampian cohort (n=193). (D) GSE87211 (n=200). Pearson correlation co-efficient plotted.
